# Clinical application of liquid biopsy in colorectal cancer: detection, prediction, and treatment monitoring

**DOI:** 10.1186/s12943-024-02063-2

**Published:** 2024-07-16

**Authors:** Xiang-Yuan Tao, Qian-Qian Li, Yong Zeng

**Affiliations:** 1 Translational Medicine Center, Hunan Cancer Hospital, The Affiliated Cancer Hospital of Xiangya School of Medicine, Central South University, Changsha, China; 2 School of Pharmacy, University of South China, Hengyang, China

**Keywords:** Colorectal cancer, Liquid biopsy, Circulating tumor cells, Circulating tumor DNA, Tumor-associated platelets, Clinical application

## Abstract

Colorectal cancer (CRC) is one of the most prevalent malignancies affecting the gastrointestinal tract and is ranked third among cancers with the highest incidence and second-highest mortality rate worldwide. CRC exhibits a slow progression providing a wide treatment window. The currently employed CRC screening methods have shown great potential to prevent CRC and reduce CRC-related morbidity and mortality. The diagnosis of CRC is achieved by colonoscopy and tissue biopsy, with studies showing that liquid biopsy is more effective in detecting and diagnosing early CRC patients. Increasing number of studies have shown that the tumor components shed into circulating blood can be detected in liquid form, and can be applied in the clinical management of CRC. Analysis of circulating tumor cells (CTCs), circulating tumor DNA (ctDNA), or tumor-associated platelets (TEPs) in the blood can be used for early screening and diagnosis of CRC, aid tumor staging, treatment response monitoring, and prediction of CRC recurrence and metastasis in a minimally invasive manner. This chapter provides an updated review of CTCs, ctDNA, and TEPs as novel biomarkers for CRC, highlighting their strengths and limitations.

## Introduction

Colorectal cancer (CRC) is a common digestive tract tumor and the second most fatal cancer globally [1–3]. Recent research has shown that poor dietary habits and lifestyle choices may increasing the morbidity and mortality rates of CRC, which continues to pose a significant threat to human health [4]. The pathogenesis of CRC originates from changes in benign or precancerous polyps which slowly transform over several years to invade the bowel wall, spread to nearby lymph nodes, or metastasize to other parts of the body. Thus, its diagnosis and treatment are also delayed [5]. Studies have indicated that the five-year survival rate of stage I CRC is 91%, but drops to 72% for locally advanced CRC and even to 14% for stage IV [6]. This suggests that early screening can effectively reduce the morbidity and mortality of CRC [7, 8]. The currently used clinical methods for detecting CRC include colonoscopy, imaging, tissue biopsy, and tumor markers. However, these methods have various limitations. For instance, imaging increases the burden of radioactive exposure and has a low recognition rate of early-stage cancer. Tissue biopsy and colonoscopy are invasive tests, and tissue biopsy can be difficult to obtain due to the intricate location of the tumor, which may cause errors in the measured results due to tumor heterogeneity. Currently, stool-based CRC screening methods are widely used. The fecal immunochemical test (FIT) is a commonly employed clinical screening method for intestinal lesions, but it has low sensitivity for CRC detection [9]. Multitarget stool biomarkers, derived from fecal sources, include DNA [10], mRNA [11], protein [12], and intestinal microbial flora detection [13]. However, these biomarkers are still in various stages of development and application. The multitarget stool biomarker test has several limitations: the unappealing nature of stool-based tests for many individuals, higher cost compared to FIT, and lower sensitivity for detecting advanced adenomas (AA) compared to colonoscopy [9]. Moreover, none of these methods can track the tumor’s dynamic changes or assess the treatment efficacy. Therefore, researchers should explore new and less invasive methods for tumor detection and predicting response to tumor therapy during the asymptomatic early stages of CRC.

**Fig. 1:**
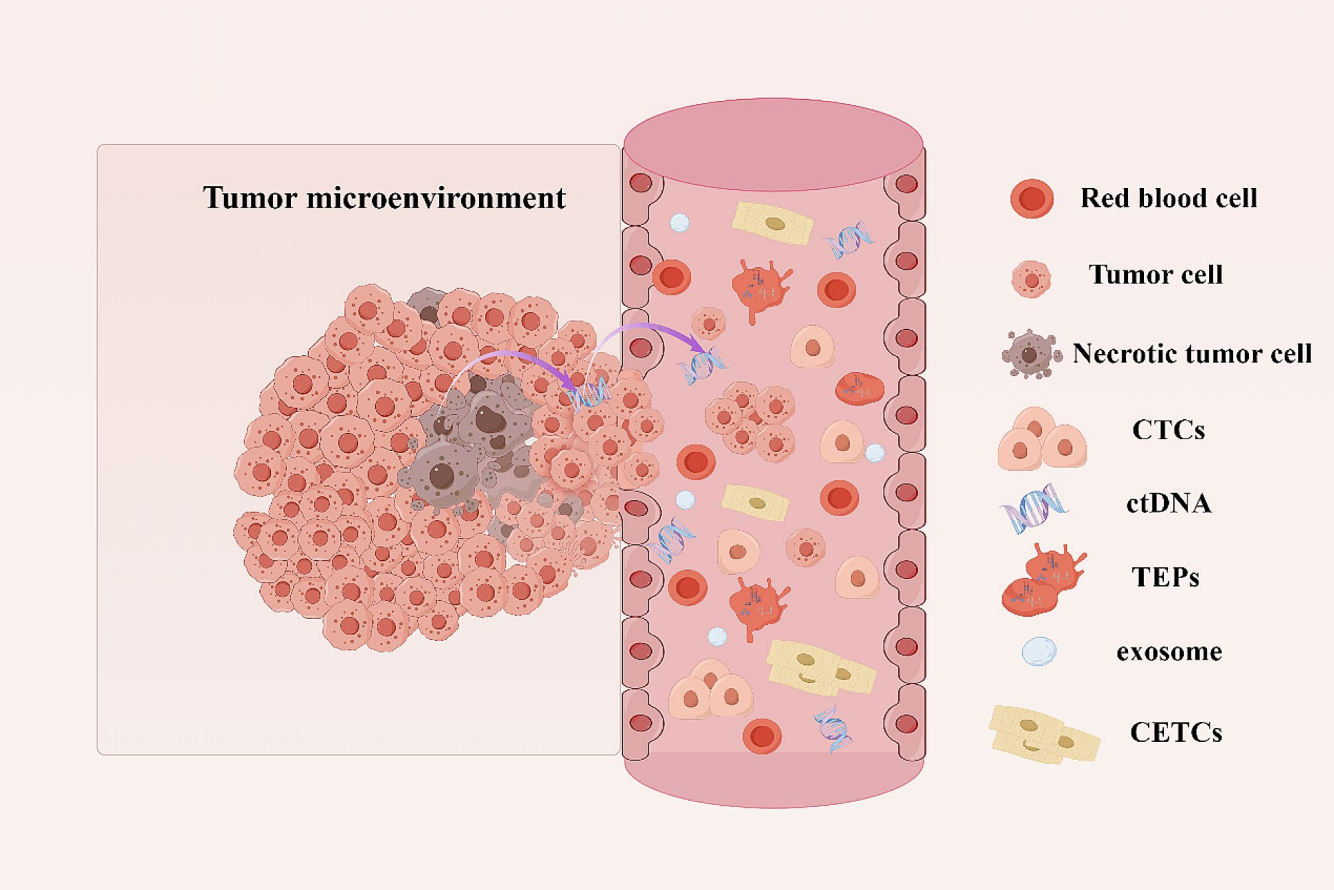
CTCs, ctDNA, and TEPs in peripheral blood. CTCs are cells that shed from the primary tumor site or recurrence site and enter the blood. With the growth of the tumor, specific changes occur in the tumor microenvironment, and some CTCs will have abnormal epithelial-mesenchymal transition (EMT) phenomena, forming circulating epithelial tumor cells (CETCs) with metastasis and invasion abilities; ctDNA comes from apoptotic and necrotic tumor cells, which release their fragmented DNA into the blood circulation and contain the same genetic information as the original tumor cells. TEPs are one of the most abundant sources of liquid biopsy and can promote the survival of tumor cells and the progression of cancer. By Figdraw

**Fig. 2:**
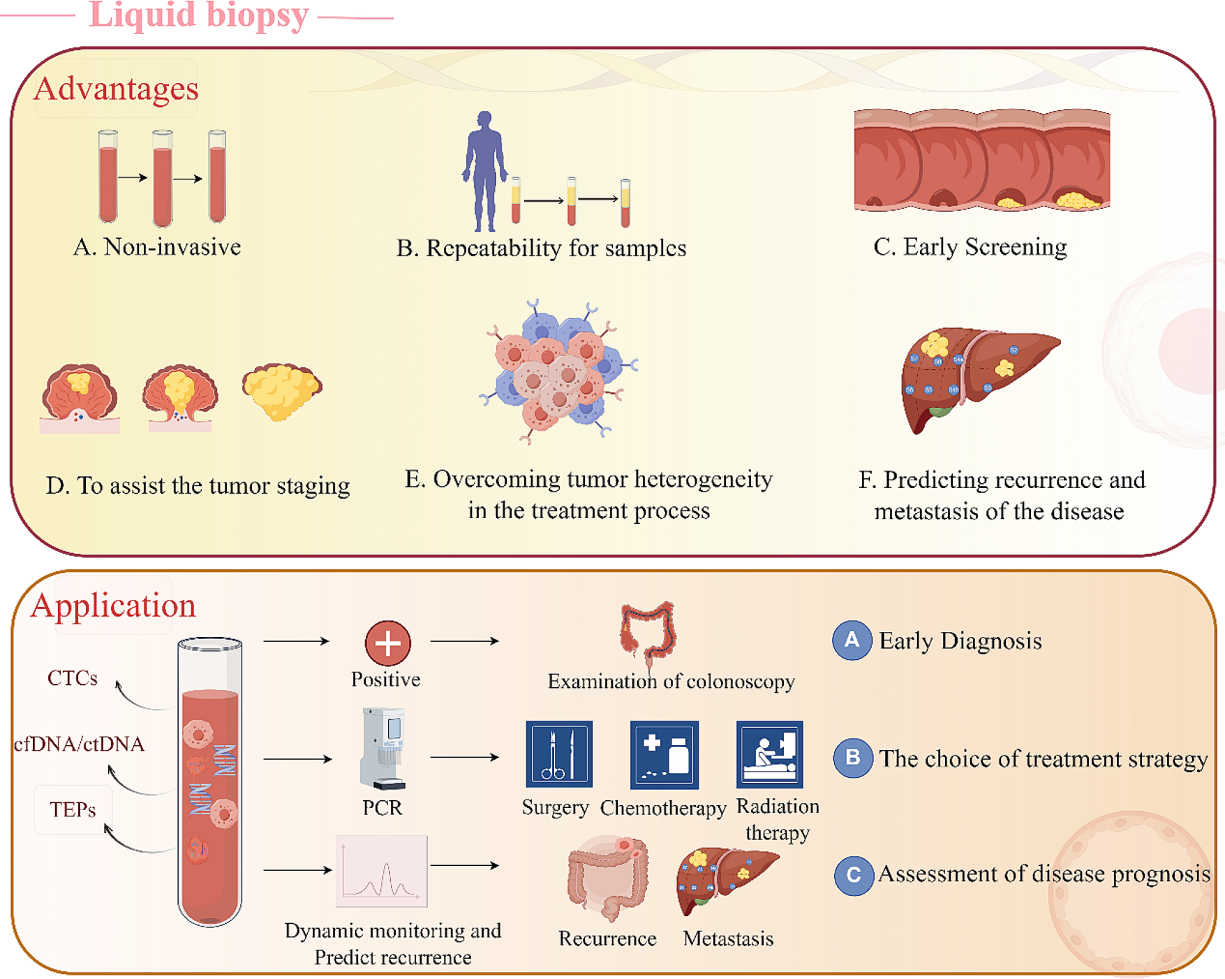
The advantages and application of liquid biopsy in CRC. The advantages of liquid biopsy mainly include non-invasiveness, repeatable sampling, effective early screening, assisting in tumor staging, and predicting the recurrence and metastasis of CRC. The application value of liquid biopsy is reflected in its ability to conduct early diagnosis of CRC, select treatment strategies, monitor treatment responses, and evaluate the prognosis of patients. By Figdraw

In recent years, scientists have developed a new detection technique named ' liquid biopsy ‘. This is a non-invasive sampling technique that can detect CTCs, ctDNA, TEPs, exosomes, and tumor marker proteins in peripheral blood or other body fluids of patients to obtain tumor-related information (Fig. 1). It is therefore useful in assisting the diagnosis and treatment of tumors making an ideal diagnostic technique in the field of “precision medicine” [14, 15]. Liquid biopsy is an alternative technology for early screening and detection of tumors. Liquid biopsy is a rapidly developing field with several advantages over traditional tissue testing. It is non-invasive, easy to perform, and enables frequent sample collection for monitoring purposes. This makes it ideal for early tumor detection, evaluating treatment response, identifying drug resistance, predicting disease recurrence and metastasis, overcoming tumor heterogeneity, and tracking genomic evolution with epigenetic analysis. Therefore, liquid biopsy is a promising modality in the management of CRC. The application of liquid biopsy in CRC facilitates early detection, post-surgery monitoring of patients with feasible resection, and selecting individualized treatment plans for those with unresectable tumors (Fig. 2). This can help slow down the progression of the disease and improve the survival rate and quality of life of patients.

This review focuses on the potential of liquid biopsy in CRC for early detection, prognosis, treatment response monitoring, and drug efficacy assessment. We explore the sources, characteristics, detection methods, and clinical applications of three key circulating tumor markers (CTCs, ctDNA, and TEPs) in CRC patients. Moreover, the limitations of the current clinical application of liquid biopsy in CRC and potential future directions are highlighted.

## Circulating tumor cells in CRC

### Circulating tumor cells

In 1869, Australian physician Thomas Ashworth made a groundbreaking discovery while observing the peripheral blood of a patient with metastatic cancer. He identified the presence of cells shed from the primary tumor or recurrent sites, circulating within the bloodstream [16]. As a result of tumor growth and changes in the surrounding microenvironment, some CTCs undergo the epithelial-mesenchymal transition (EMT). This transformation transforms CTCs into circulating tumor-derived endothelial cells (CETCs), which can metastasize and invade other parts of the body. CETCs can acquire stem cell-like traits, enhancing their survival by reducing apoptosis and senescence. Their presence also promotes immunosuppression. These CTCs detach from the primary tumor and travel through the bloodstream or lymphatic system. Upon reaching their destination, they undergo mesenchymal-epithelial transition (MET), regaining epithelial cell characteristics to form metastases and proliferate uncontrollably [17]. Considering that CTCs can spread cancer to other organs through the bloodstream, we hypothesize that their ability to infect organs remotely may be a mechanism through which they influence cancer metastasis [18].

### CTCs enrichment and detection

Generally, CTCs are produced in very low concentrations in peripheral blood, usually ranging from 1 to 10 cells per 10 mL of blood. Moreover, they have a short half-life of about 1–2.5 h, making them difficult to detect [19]. Therefore, CTCs can be detected using the following two methods: the immunogenicity method and the unique physical (size, density, etc.) method based on CTCs. The immunogenic isolation enrichment methods can be further classified into positive and negative enrichment depending on the target of isolation. The most commonly used membrane protein in the positive enrichment process of CTCs is epithelial cell adhesion (EpCAM) [20]. CellSearch employs EpCAM to identify CTCs, and is currently the only method approved by the US Food and Drug Administration for this purpose [21, 22]. Recent studies have indicated that mesenchymal CTCs (M-CTCs), which are more invasive and metastatic than epithelial CTCs, cannot be detected using EpCAM-based enrichment methods [23]. Another method for isolating CTCs leverages the size difference between cancer cells and non-malignant blood cells. This technique, called Isolation by Size of Epithelial Tumor Cells (ISET), relies on biophysical properties to enrich the sample for potential cancer cells. ISET separates CTCs based on biophysical differences between cancer cells and non-malignant blood cells. Compared with CellSearch, the ISET filtration system can process larger samples and separate more CTCs for subsequent functional or genomic analysis [22]. In summary, there is a need to update the separation and capture techniques of CTCs and improve their enrichment efficiency and purity.

**Fig. 3:**
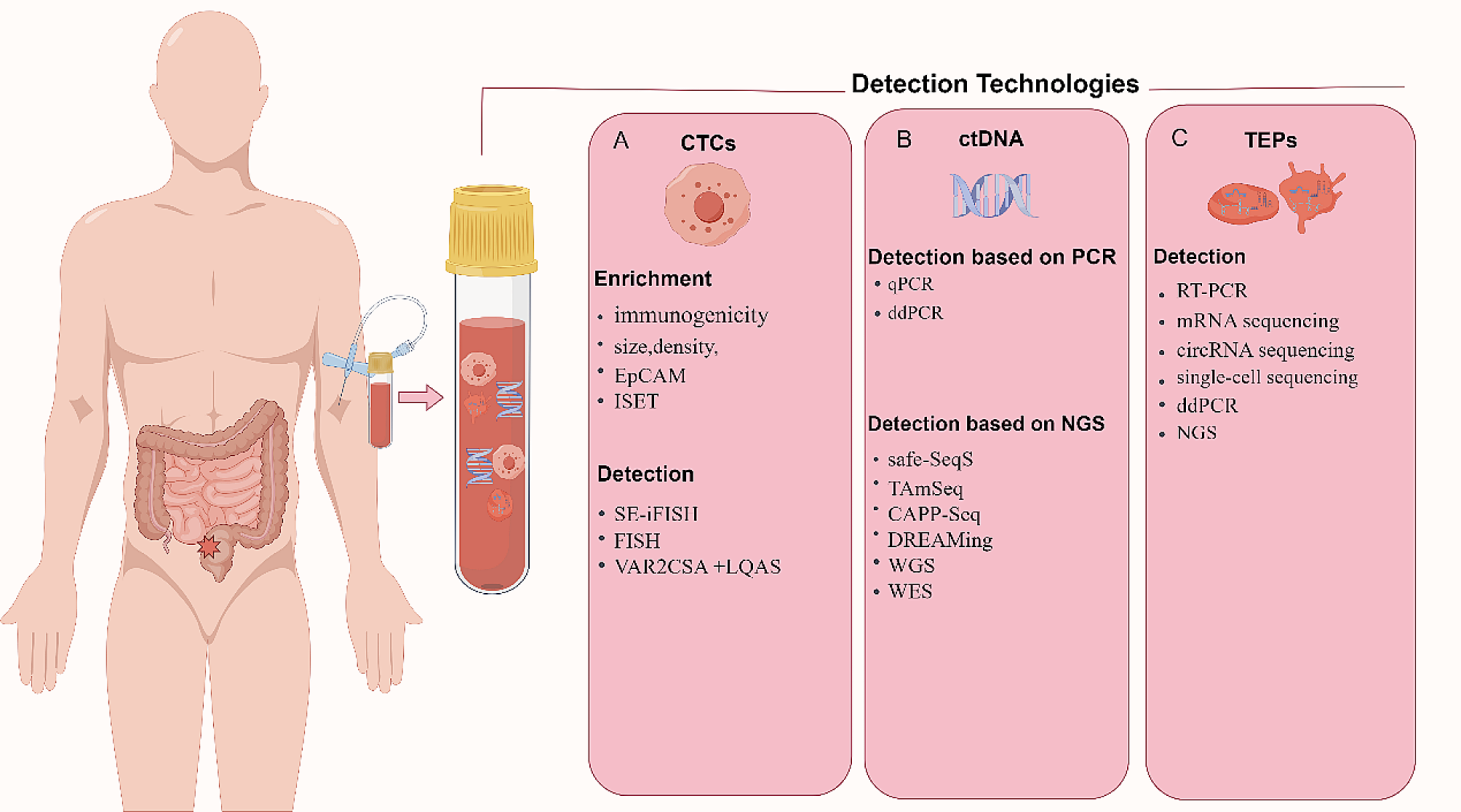
Various detection methods of CTCs, ctDNA and TEPs in liquid biopsy. By Figdraw

In 1998, Racila used immunomagnetic enrichment with ferromagnetic fluid-coupled antibodies against EpCAM. By combining this method with flow cytometry, the detection of CTCs was achieved. Sara R. et al. bound recombinant malaria VAR2CSA protein (rVAR2) to magnetic bead-coupled cancer cells to detect enriched CTCs. Highly sensitive capture methods like rVAR2 can improve CTC detection without reducing accuracy, as shown in previous research [19]. Moreover, some methods, such as CTCs SE-iFISH technology, negate the need for epithelial markers. This technique employs subtractive enrichment (SE) followed by immunostaining and fluorescence in situ hybridization (FISH) for CTC identification. CD45 immunostaining and DAPI staining are then used to characterize the identified CTCs [24, 25] (Fig. 3A).

### CTCs can be used for early screening and diagnosis of CRC

Early diagnosis of CRC is crucial for improved survival. Patients diagnosed at stage I or II have a significantly higher survival rate, with up to 90% surviving for 5 years or more. In contrast, the survival rate for patients diagnosed at stage III or IV drops dramatically to only 13%. This highlights the significance of early screening and diagnosis of CRC in improving patient outcomes [26]. The traditional methods for detecting CRC include carcinoembryonic antigen (CEA), computed tomography, and colonoscopy. These methods have various limitations [27, 28]. For instance, although colonoscopy is the “gold standard” for early detection of CRC, it is highly invasive, which decreases patient compliance [29]. Through a survey of 109 individuals who refused colonoscopy, Andreas Adler et al. found that 97% of them were willing to undergo non-invasive examinations, indicating that non-invasive examinations may improve patient compliance [30]. It is reasonable that this might very well be the situation, but in small data sets, there are indeed selection biases and the data is somewhat difficult to be generalized. In recent research suggests that CTCs offer potential as a non-invasive screening and diagnostic tool for early-stage CRC. This approach could improve patient compliance and address the challenge of low colonoscopy adherence [15]. Wen-Sy Tsai et al. compared the levels of CTCs in the blood of 667 subjects before undergoing colonoscopy and the operation. They found that 307 out of 325 CRC patients were effectively identified by CTCs testing with a sensitivity of 95.2% (95% confidence interval [CI]:92.2-97.3%). This implies that CTCs testing is highly concordant and can facilitate early diagnosis [31]. Another study found that 41 blind samples had a 90.0% accuracy in the detection of CTCs by flow cytometry, with 80.0% sensitivity and 100.0% specificity [32]. In 2021, researchers combined the analysis of CTC counts with CEA and found that this approach enhanced the accuracy of CRC detection. Moreover, the joint assessment of CTC counts and immunochemical fecal occult blood tests (iFOBTs) can potentially reduce false-positive results on iFOBTs [33]. Therefore, combining the use of CTCs with other non-invasive tests may be a promising solution to address this issue of low compliance with colonoscopy testing. Although initial research on CTCs for CRC has demonstrated promising findings, current studies are limited by small sample sizes. Larger-scale studies and robust clinical evidence are still needed to definitively establish the effectiveness of CTCs as a pre-colonoscopy screening tool for early detection of CRC. In the early-stage CRC, it is difficult to detect CTCs, and different assays have shown varying accuracy. Dong Hoon Baek et al. detected CTCs in 88 CRC patients and 31 healthy volunteers using a centrifugal microfluidic system coupled with a new fluid-assisted separation technology (FAST). When the critical value of CTCs was set to 5/7.5 mL of blood using absolute value as a criterion, the sensitivity and specificity for distinguishing CRC patients from healthy controls were 75% and 100%, respectively [34]. Wen-Sy Tsai et al. isolated and detected CTCs using the CellMax platform, and employed the mean of CTC counts (healthy subjects: 2.11 CTCs, CRC patients: 16.99 CTCs, *P* < 0.0001) as a criterion for scoring. They obtained a specificity of 82.1% and sensitivity of 89% in 235 subjects [31]. In another testing experiment based on the size of CTCs, the percentage of CTCs-positive samples per 5 ml of blood was 44.4% about the mean value of cancer stage and CTCs. Another study analyzed and counted CTCs in 76 cancer patients and 20 healthy subjects. They found that CTCs were detected in 50 patients, 55.0% in stage II, 68.8% in stage III, and 86.7% in stage IV [35]. In this study, they utilized the mean and relative changes in CTC counts to represent the number of CTCs at different stages of CRC. The higher the content of CTCs, the higher the graded stage of CRC. In conclusion, these three experiments demonstrate that the unity of detection methods and standards can be achieved while ensuring the accuracy of CTC detection of CRC. Future studies are needed to develop optimized methods.

### CTCs for CRC treatment monitoring and efficacy assessment

Currently, the most effective treatment for CRC is surgery combined with chemotherapy [36]. Following surgery, approximately 20% of stage II patients and 35% of stage III patients experience a relapse within five years [37]. Even after surgery, microscopic tumor remnants called minimal residual disease (MRD), can persist in the body. This MRD is a major contributor to poor treatment outcomes, often leading to tumor recurrence and metastasis [38]. The current methods of evaluating post-surgery treatment are based on imaging, which requires a long period of treatment for 10–12 weeks [39], CTCs provide a real-time biopsy that can dynamically monitor changes in CTCs in the blood. Therefore, many researchers combine the surgical treatment evaluation of CTCs and CRC. For instance, Rosa Divella et al. used an immunomagnetic separation method to isolate CTCs from peripheral blood and concluded that CTCs can be classified as single cells or clusters. For single CTCs, a low content was considered 27 or fewer cells, while a high content was considered 47 or more cells. In contrast, the number of CTCs in clusters was determined by the combined total of individual CTCs within the cluster, with each cluster containing between 3 and 25 CTCs. [40]. Elsewhere, researchers identified potential CTCs based on the size of the nucleus (> 20 μm), High nuclear mass ratio, irregularity of the nuclear membrane, and the density of the nucleus by the flow cytometry counting method. Their CTC counts were evaluated by different criteria from those of the immunomagnetic isolation method, with donors with malignant disease (*n* = 30) and donors without malignant disease (*n* = 10) [41]. Therefore, for CTCs to be truly used in the clinic for the assessment of MRD after CRC surgery, standardized techniques for the identification and detection of CTCs should be established. Moreover, the criteria for assessing the counts of CTCs need to be formulated and validated.

CTCs are non-invasive biopsies that can be utilized to guide targeted drug selection for cancer treatment by evaluating patient drug sensitivity. Recently, Enrique Aranda et al. performed a randomized phase III VISNÚ-1 trial comparing 5-fluorouracil/glyceryl folinic acid, oxaliplatin, irinotecan (FOLFOXIRI) in combination with bevacizumab versus oxaliplatin, leucovorin, 5-fluorouracil (FOLFOX) -bevacizumab for the treatment of metastatic colorectal cancer. They found a median progression-free survival (PFS) of 12.4 months (95% CI 11.2 to 14.0) for FOLFOXIRI bevacizumab and 9.3 months (95% CI 8.5 to 10.7) for FOLFOX bevacizumab (stratified HR 0.64; 95% CI 0.49 to 0.82; *P* = 0.0006). Their findings indicate that CTC counts can improve the selection of patients who are suitable for intensive first-line therapy [42]. However, the concentration of CTCs in peripheral blood is too low, so the establishment of CTCs in vitro culture from individual patients should be considered as an approach for predicting potential drug resistance and sensitivity of patients [43]. In 2017, Grillet et al. established stable CTC cell lines CTC41, CTC44, and CTC45 derived from patients with advanced metastatic CRC. These three CTC cell lines were tested for drug sensitivity to the standard chemotherapeutic regimens 5-fluorouracil and SN-38 (the active metabolite of irinotecan) compared to cells derived from primary CRC. The study demonstrated that metastatic CTC lines showed high expression of genes associated with irinotecan resistance, suggesting that in vitro culture and characterization of CTCs may be an attractive platform for non-invasive monitoring of changes in drug sensitivity when new mutations occur in tumors [44]. However, cell lines evolve during culture, and the extent of genetic and transcriptional heterogeneity that arises as well as its functional consequences need to be investigated [45]. In addition, CTCs allow tumor genome sequencing to identify unknown changes or mutations, and select drug types based on the gene expression profile of CTCs. Moreover, in vitro amplification platforms can be used to increase the obtainable amount of CTCs. This integration with other technologies allows for drug selection and efficacy testing, even with initially low CTC counts. In addition, 5/7.5 mL of CTCs were previously detected in patients receiving metastatic cancer treatment, and this can predict the therapeutic effect. The survival duration of patients with reduced CTCs levels is significantly higher compared with that of patients with elevated CTCs levels (17.67 ± 5.90 months vs.4.53 ± 0.54 months, *P* < 0.001) [46]. Rosa Divella et al. observed that the number of CTCs per milliliter of blood in 7 patients with metastatic colorectal cancer (mCRC) was decreased from 17 (± 7.528) before treatment with FOLFOX to 6 (± 4.46 ) after treatment, indicating that CTCs serve as non-invasive real-time biopsy for assessing the therapeutic effect of drugs on the disease [41]. However, these results are based on a small cohort, and large-scale studies are needed to verify the clinical value of CTCs in evaluating drug efficacy.

### CTCs for the prognosis of CRC

Although radical surgery can treat some patients, those with heavy tumor burden, neurovascular invasion, and distant metastasis still experience a poor prognosis. Metastasis is the main cause of poor CRC prognosis [47]. CTCs are cells that fall off from primary tumors or recurrent sites and enter blood circulation. Therefore, the number of CTCs can predict PFS and overall survival (OS) in CRC patients [48]. As early as 2008, by setting the baseline CTCs to 3/7.5mL in 430 mCRC patients, the PFS and OS of patients above the baseline were shorter (PFS: 4.5 vs. 7.9 months; *P* = 0.0002; OS: 9.4 vs. 18.5 months; *P* < 0.0001) [49]. Recent meta-analyses demonstrate a strong correlation between the presence of CTC) and poor survival rates in CRC patients. This includes reduced overall survival (OS: HR 2.36, 95% CI: [1.87–2.97]; *P* = 0.006) and a higher risk of aggressive disease progression (PFS: HR 1.83, 95% CI: [1.42–2.36]; *P* < 0.00001). These findings suggest that the presence of CTCs serves as a reliable independent predictor of unfavorable outcomes in CRC [50].

Similar to primary tumors, CTCs are heterogeneous and characterized as clusters and single cells composed of phenotypically and genetically different subsets, which lead to more aggressive and metastatic phenotype of CTCs [51, 52]. In addition, studies have shown that CTCs will change with the growth of tumors and the microenvironment. Some CTCs may undergo abnormal EMT and transformation into CETCs, acquiring stem cell characteristics, reducing apoptosis and senescence, and promoting immunosuppression so that CTCs can escape anti-metastasis checkpoints, mediate distant metastasis, and enhance CTCs invasion [17, 53]. The diverse characteristics of CTCs, partly attributed to the EMT process, present an opportunity to predict CRC metastasis. By analyzing the EMT status and specific markers associated with EMT in CTCs, we may gain valuable insights for predicting metastatic potential. In a recent study, Xiao-Cui Hong et al. identified CTCs subsets based on EMT phenotype and the expression levels of regenerating liver-3 (PRL-3) and matrix metalloproteinases-9 (MMP9) in CTCs in 172 patients. Their results showed that patients with positive expression of PRL-3 and MMP9 in CTCs had significantly lower PFS (*P* = 0.0024) and OS (*P* = 0.095) relative to those with negative expression of PRL-3 or MMP9. Finally, multivariate Cox analysis revealed that the positive expression of PRL-3 and MMP9 in CTCs may be an independent prognostic factor for PFS deterioration [54]. Moreover, evidence suggests that interstitial CTCs reflect enhanced malignant diseases and metastatic potential in CRC. Guang Lu et al. conducted multiple EMT-related CTC tests and follow-ups on 101 patients who underwent radical surgery and chemotherapy for CRC. They reported increased total number of CTCs (*P* < 0.01), epithelial CTCs (E-CTCs) (*P* = 0.032), and increased number of E-CTCs (*P* = 0.015), which were significantly correlated with the PFS rate in patients [55]. Similarly, J. Hou et al. examined peripheral blood samples from 51 CRC patients before anti-tumor therapy and found that patients with M-CTCs exhibited significantly shorter PFS compared with those without M-CTCs (15 vs. 26 months, *P* = 0.011), and the presence of M-CTCs was the only independent prognostic factor for poor PFS [56]. While CTCs cannot replace traditional methods like tumor markers and imaging for pinpointing the exact location of recurrence or metastasis in CRC after surgery, they can inform clinical decision-making. Analyzing CTCs can potentially help determine the frequency of these other tests, either reducing their use or extending the time between examinations. Moreover, combining CTC results with other tumor markers holds promise for improving overall diagnostic accuracy [48].

## Circulating tumor DNA in CRC

### Circulating tumor DNA

In 1948, Mandel and Métáis made a discovery that cell-free DNA (cfDNA) could be detected in the blood of cancer patients [57]. cfDNA is a snapshot of dying cells throughout the body, consisting of double-stranded DNA fragments that are continuously cleared from the bloodstream. Generally, cfDNA is derived from normal healthy white blood cells and stromal cells. However, cfDNA can also incorporate DNA from circulating tumor cells released into the bloodstream during apoptosis or necrosis in cancer patients. This DNA is referred to as ctDNA [58]. A study published in 1999 found that abnormal methylation, a potential indicator of cancer, was present in tumor tissues from 16 out of 22 patients with non-small cell lung cancer. This finding suggests that ctDNA, which carries similar methylated patterns, might be useful for detecting and monitoring cancer recurrence [59]. ctDNA provides a more comprehensive overview of the mutational spectrum present in a patient’s tumor compared with a snapshot of a single tissue biopsy. It not only allows for sequential sampling in a completely non-invasive manner but also enables the analysis of specific tumor types in specific anatomical locations in greater detail [60]. Numerous studies on ctDNA have shown that it can be used, in combination with next-generation sequencing (NGS), as a surrogate substrate for tumor tissues to detect mutations and diagnose CRC [61]. ctDNA holds significant potential for various applications in CRC management. It can be used to detect gene mutations, potentially aiding in early diagnosis, analyzing the molecular makeup of the tumor, and predicting how patients might respond to treatment. Moreover, ctDNA can help monitor the risk of cancer recurrence months before it becomes visible through medical imaging. However, it is important to note that ctDNA levels can vary significantly within and between different tumor types [62]. Despite this limitation, ctDNA is emerging as a valuable biomarker for predicting a patient’s prognosis and tailoring treatment plans in personalized CRC management.

### Methodology of ctDNA

ctDNA is a component of cfDNA that is shed from cancer cells into the blood [62, 63]. The detection techniques for ctDNA are classified into two. One is a simple classification based on whether it involves polymerase chain reaction (PCR) or NGS, and the other is based on the genomic coverage of ctDNA components in plasma, which is divided into targeted and non-targeted methods [64]. Initially, real-time quantitative PCR (qPCR) technology was primarily used for ctDNA detection, but its ctDNA extraction method could not concentrate low-concentration DNA and required multiple samples, so it was mainly applied in advanced patients with high DNA content. Recent research has shown that magnetic ionic liquids (MILs) offer a promising technique for ctDNA analysis in early-stage cancer [66]. MILs can rapidly pre-concentrate DNA from blood plasma and be directly incorporated into various qPCR buffers, streamlining the process. This is particularly valuable as traditional qPCR methods can only detect mutations present in over 10% of mutant allele fraction (MAF), whereas droplet digital PCR (ddPCR) offers significantly higher sensitivity (Sn), detecting mutations present in as little as 0.1% of the DNA. Its detection specificity (Sp) and Limit of Detection (LoD) can reach 98% and 0.001–0.01%, respectively [65]. Although PCR is fast and inexpensive, it is one of the most commonly used techniques for detecting ctDNA, but it cannot efficiently detect unknown mutations. In recent years, another method based on NGS, which can screen unknown variants without high-throughput properties, has been widely adopted for the detection of ctDNA. Currently, NGS can detect less than 1% of MAF. The application of targeted DNA mainly includes a safe sequencing system (safe-SeqS) and targeted analysis of the methylome sequencing (TAmSeq) [66], cancer personalized profiling by deep sequencing (CAPP-Seq), and bead, emulsion, amplification, and magnetics (BEAMing) technology has been performed by deep sequencing [64]. Moreover, NGS can also perform non-targeted DNA technology and genome-wide analysis of copy number variations (CNAs), point mutations, and other genetic variations through whole-genome sequencing (WGS) or whole-exome sequencing (WES), to identify unknown aberrations [66]. Genome-wide ctDNA analysis faces several limitations in clinical settings. It often requires larger blood samples, higher ctDNA concentrations in the blood, and achieves a lower overall detection sensitivity (often exceeding 1–5%). These limitations restrict its application in patients without metastatic disease, especially for early detection of recurrence [69]. For ctDNA analysis to be widely used in clinical practice, technological advancements are needed to improve factors like target range, sensitivity, specificity, sample size requirements, and cost (Fig. 3B).

### ctDNA can be used for early detection of CRC

ctDNA is a DNA fragment released from apoptotic or necrotic cancer cells into the circulatory system. They carry somatic mutations in the tumor, and its abundance is influenced by the tumor size and stage [67]. Several studies on ctDNA analysis have demonstrated its clinical application in early screening of CRC. Abnormal DNA methylation is one of the most common epigenetic changes leading to tumor formation and is a feature of most solid cancers [68]. Huiyan Luo et al. found that using a single ctDNA methylation marker cg10673833 to detect CRC and precancerous lesions had high Sn (89.7%) and high Sp (86.8%) through a prospective non-randomized study involving 1493 participants. They reported that ctDNA methylation is a robust biomarker for early diagnosis and monitoring of CRC [69]. In addition, other scholars have indicated that abnormal DNA methylation can serve as a marker for early detection of CRC (Table 1).

The tumor suppressor gene SEPT9 has been extensively investigated in CRC and is the first blood-based test method ever reported [70, 71]. It has also been demonstrated that using different algorithms can improve the Sn and Sp of SEPT9 [72–75]. In a study of 104 CRC patients and 130 patients with colorectal polyps, a gene panel detected the methylation of SDC2 and BCAT1 in peripheral plasma samples using real-time PCR. The ctDNA methylation level and a comprehensive score of SDC2 and BCTA1 in the CRC group were significantly higher relative to those in the colorectal polyp group (6.1%, 4.4%, and 83.7%, 82.7%, respectively) [76]. Hui Li et al. used SFRP2 to detect the methylation status of SFRP2 in 17 cases of cancer tissues and paired adjacent tissues based on quantitative methylation-specific (PCR) assay. The results indicated that the methylation level of SFRP2 in 94.1% (16/17) of CRC tissues was higher compared with that in adjacent tissues (*P* < 0.001) [77].

Combining different biomarkers is a powerful approach to improve the accuracy of cancer detection and screening. This strategy has been shown to increase sensitivity and specificity. For instance, combining the detection of two markers, SEPT9 and SDC2, led to a significant increase in both sensitivity (from 67 to 86.5%) and specificity (from 89 to 92.1%) for diagnosing CRC. This finding highlights the potential of combining methylated SEPT9 and SDC2 detection as a reliable approach for CRC diagnosis [75, 78]. Similarly, in a prospective cohort study involving more than 2000 patients, the detection of BCAT1 and IKZF1 gene methylation in cfDNA showed that Sn was 66% and Sp was 94% for CRC detection [15, 79, 80]. Studies have shown that combining methylation markers in ctDNA, such as SFRP1, SFRP2, SDC2, and PRIMA1, can significantly improve the accuracy of CRC diagnosis, with sensitivity exceeding 90% in some cases [84]. While ctDNA holds promise as a biomarker for early CRC detection, its low concentration in the body necessitates highly sensitive detection technologies.

**Table 1:** Markers of ctDNA methylation in early detection of CRC

Marker	Sample types	Sensitivity	Specificity	AUC	Reference
SEPT9	plasma	61.22%	93.7%	-	[73]
SEPT9 SDC2	Plasma plasma	67% 76.9%	89% 90.8%	0.87 0.855	[75] [76]
BCAT1	plasma	83.7%	93.9%	0.908	[76]
SFRP2	serum	69.4%	87.35%	-	[77]
VIM	serum	59.0-90.7%	72.5-93.0%	-	[79]
TPEF	serum	65.0-81.0%	69.0-90.0%	-	[79]
MYO1-G	plasma	84.3%	94.5%	0.94	[78]
IKFZ BCAT1	plasma	62.1-95.0%	92.0-95.0%	-	[15, 79]
IKFZ BCAT1	plasma	66%	-	-	[80]
SEPT9 SDC2	serum	86.5%	92.10%	-	[75]
SFRP1 SFRP2	plasma	91.5%	97.3%	0.978	[81]

“-” Sensitivity and specificity values not mentioned in the original study; AUC: area under ROC curve

### ctDNA is used for treatment monitoring and efficacy evaluation of CRC

ctDNA has been applied in the management of various diseases: as a treatment response monitoring tool, and as a predictive biomarker for treatment selection. It is used for MRD detection, early CRC recurrence monitoring, molecular mapping, and prediction of treatment response [82]. For example, a prospective study based on NGS analysis showed that ctDNA could monitor MRD in 230 patients with stage II CRC cancer. Among 178 patients who did not receive adjuvant chemotherapy, ctDNA was detected in 11 patients (7.9%) after surgery. Notably, the recurrence rate of ctDNA-positive patients was higher than that of ctDNA-negative patients after a median follow-up of 27 months (HR 18,95% CI 7.9–40; *P* < 0.001) [83]. Another prospective study of investigating ctDNA detection by ddPCR and mass spectrometry-based platform MassARRAY ^®^ showed that ctDNA can be used as a marker for MRD after CRC treatment [84]. The study by Jiaolin Zhou et al. investigated the potential of ctDNA for several purposes in patients with locally advanced rectal cancer (LARC): predicting response to neoadjuvant chemotherapy (nCRT), monitoring tumor burden, and predicting survival rates. Their findings suggest that ctDNA may serve as a real-time indicator for accurately reflecting tumor burden [85].

The half-life of ctDNA is short, usually 20–60 min, thus the quantitative change of ctDNA can reflect the progress of the disease and can be used to monitor CRC and evaluate the therapeutic effect of treatments [63]. For example, Feng Wang et al. collected blood samples from 171 patients with mCRC. It was observed that patients with plasma RAS/BRAF clearance had better PFS and OS than those with RAS/BRAF mutations [86]. In addition, ctDNA was utilized to explore whether the dose of adjuvant chemotherapy can be reduced without increasing the risk of recurrence by analyzing ctDNA in patients with stage II CRC. The 3-year PFS rate was 86.4% in ctDNA-positive patients who received adjuvant chemotherapy, and 92.5% in ctDNA-negative patients who did not receive adjuvant chemotherapy, indicating that ctDNA may serve as a guide for reducing adjuvant chemotherapy without affecting PFS [87]. Cetuximab is the main targeted therapy for advanced colorectal cancer. It inhibits the growth of tumor cells and blood vessels by binding to epidermal growth factor receptor (EGFR) and stimulates immune cells to produce strong anti-tumor effects. However, in CRC patients with RAS and BRAF mutations which will lead to the resistance of this type of patients to cetuximab, and the treatment effect and prognosis are poor [88]. Mechanistically, ctDNA detection results showed that the acquired resistance of cetuximab in CRC patients was not only caused by genetic resistance drivers, but could be induced by several other factors. For instance, stromal remodeling is a non-genetic resistance mechanism driving cetuximab resistance [89]. Khan KH et al. explored the development of mCRC resistance to cetuximab through a combination of liquid biopsy and mathematical modeling approaches to track the RAS pathway abnormalities involving the cfDNA. They found that a higher frequence of cfDNA sampling increased the accuracy of predicting the time to disease progression and provided a window of opportunity for therapeutic intervention [90]. In addition, ctDNA may serve as a biomarker for examining the efficacy of regorafenib in treating mCRC patients. In a phase II trial, dynamic contrast-enhanced magnetic resonance imaging (MRI) was performed on RAS mutant mCRC patients before and 15 days after treatment, and ctDNA was collected monthly to detect RAS clonal mutations. It was found that patients with reduced RAS clonal mutations in ctDNA had better PFS (HR 0.21 (95% CI 0.06 to 0.71), *P* = 0.01) and OS (HR 0.28 (95% CI 0.07–1.04), *P* = 0.06) [91]. Although ctDNA can be used to monitor treatment efficacy in CRC patients, it has some limitations. Previously, it was found that patient-derived organoids (PDOs) could predict patient’s response to targeted drugs when cancer cells derived from cancer patients are cultured in vitro [92]. Therefore, if ctDNA is applied in combination with some new detection technologies, it will enhance the therapeutic effect and optimize individualized treatment. In conclusion, evidence suggest that ctDNA can be used to monitor CRC disease progression, guide clinical treatment, and provide new opportunities for personalized therapy. However, when ctDNA is incorporated into clinical practice, it is crucial to reach a consensus on standardized testing procedures and unified result evaluation criteria.

Tumor immunotherapies have received much attention in recent years, and immunotherapy is now clearly effective in treating CRC patients with mismatch repair-deficient (dMMR)/microsatellite instability-high (MSI-H). This is caused by the high immunogenicity and extensive T-cell infiltration of dMMR/MSI-H tumor tissues, which increases their sensitivity to programmed cell death protein 1 (PD-1)/ programmed cell death protein ligand 1 (PDL-1) inhibitor therapy with a better clinical response [93, 94]. The NCCN guidelines of colorectal cancer (Version 3.2024) emphasize a promising development: mutations in DNA polymerase epsilon (POLE) or DNA polymerase delta 1 (POLD1) genes. These mutations in CRC patients may indicate a potentially better response to immunotherapy due to enhanced immune stimulation [95]. Studies have shown that mCRC patients with POLE/POLD1 mutations can achieve relatively good therapeutic effects when using immune checkpoint inhibitors (ICIs), and POLE/POLD1 is an independent predictor that can be used to predict the efficacy of immunotherapy [96].

Currently, doctors rely on tissue biopsies to determine a patient’s mismatch repair (MMR)/microsatellite instability (MSI) and POLE/POLD1 status. However, obtaining tissue samples can be complex, and it is often necessary to monitor these markers throughout treatment. Therefore, developing a method that uses liquid biopsies (such as blood tests) for these tests would be highly valuable. Construction of the above methodology should be further optimized as follows: (1) Design of gene panel; (2) Consistency of results between liquid and tissue samples; (3) Setting cut-off for immunization drug selection and dynamic treatment monitoring and a range of other technical issues. To fully validate the methodologies mentioned above, further research involving clinical studies with a significant number of participants is necessary.

### ctDNA may be a prognostic marker of CRC

In 2002, a small study in France demonstrated that ctDNA may be a prognostic marker for CRC patients [97]. They reported that analysis of postoperative ctDNA in CRC patients can help to predict MRD. Lone V Schøler et al. used large-scale parallel sequencing to identify somatic mutations and used it as a ctDNA marker. It was found that ctDNA could be detected in 14 of 45 patients with recurrence after surgery, but not in patients without recurrence. In addition, in 23 patients with liver metastasis, ctDNA score at 3 months after surgery could predict cancer recurrence (HR 4.9; 95% CI, 1.5–15.7; *P* = 0.007). These findings suggest that the detection of postoperative ctDNA provides MRD evidence and may guide the postoperative management of CRC patients [98]. Similar results were obtained in another prospective multicenter study, which found that positive ctDNA on day 3–7 after surgery was significantly associated with the risk of recurrence (hazard ratio [HR] = 10.98); 95% CI was 5.31–22.72; *P* < 0.001). Even after accounting for other established risk factors, the presence of ctDNA remains the strongest and most independent predictor of PFS in CRC patients. Studies show that ctDNA analysis is highly accurate in detecting recurrence, with an overall accuracy of 92.0% [99, 100].

Finally, ctDNA can predict the recurrence of CRC and the recurrence of CRC by an average of 9.4 months compared with tomography imaging [98]. The combination of traditional cross-sectional/functional imaging and ctDNA can improve the prognosis monitoring of patients after CRC resection. These observations need to be further validated in larger studies [101].

## Tumor-associated platelets in CRC

### Tumor-associated platelets

Platelets are tiny, cell-like fragments shed from megakaryocytes in the bone marrow. They are the second most abundant cell type in your bloodstream and play a crucial role in forming blood clots to stop bleeding and healing wounds [102]. Platelets in tumor patients can recognize the interaction between other cells and adjacent platelets. This allows tumor cells to interact with platelets, which enhances cancer progression by activating platelets, releasing various factors, and altering the RNA profiles of tumor cells [103]. In addition, platelets can respond to external signals, intake proteins, and nucleic acids, and change their megakaryocyte-derived transcripts and proteins, known as TEPs, making them one of the most abundant sources of liquid biopsy [104]. Studies have demonstrated the potential of TEPs as a promising tool for cancer diagnosis. In 2015, a study by Myron G. Best et al. performed mRNA sequencing on TEPs from 283 patients, successfully distinguishing individuals with localized and metastatic tumors (228) from healthy individuals (55) with 96% accuracy [100]. Building on this, Linlin Xue et al. in 2018 investigated the presence, location, and molecular characteristics of cancer by analyzing the RNA of TEPs using 20 potential biomarkers specific to non-small cell lung cancer [105]. In 2023, it was reported that TEPs are produced due to changes in intravascular platelets in the tumor microenvironment (TME), and they transform into TEPs, supporting cancer development, angiogenesis, and metastasis through degranulation and the release of microbial molecules [106]. TEPs provide several advantages in liquid biopsy: they interact directly with tumor cells, are abundant in the bloodstream, and can be easily isolated using simple centrifugation. In addition, their RNA content can be readily purified, making it ideal for detecting gene fusion and splicing variations that are indicative of cancer [107]. With the rapid development of technology, TEPs are expected to revolutionize the diagnosis, monitoring, and prognosis prediction of cancer [104].

### Methodology of TEPs

The main traditional methods used for detection of TEPs are RNA sequencing technology and reverse transcription-polymerase chain reaction (RT-PCR). Myron G. Best et al. developed the ThromboSeq platform in 2015, which uses NGS RNA-seq to detect biomarkers in TEPs and can be applied in clinical settings [108]. In 2017, they found that particle-swarm optimization (PSO)-enhanced algorithms could effectively select RNA biomarker groups (*n* = 779) from platelet RNA sequencing libraries, and accurately detect early and advanced non-small cell lung cancer (NSCLC) based on TEPs mRNA sequencing [109]. In 2019, Myron G Best et al. found that PSO-enhanced algorithms can automatically select the RNA that contributes the most to the biomarker panel to achieve RNA sequencing and cancer classification [110]. In 2021, imPlatelet classifiers were established with the ability to convert RNA sequencing data in TEPs into images, where each pixel corresponds to the expression level of a gene. In the case of a limited number of samples, deep-learning images can also accurately identify cancer [111]. In addition to sequencing the mRNA of TEPs, circRNA can also be labeled and sequenced. It has been shown that the expression of circNRIP1 in platelets is significantly down-regulated in NSCLC patients [112]. Human platelets contain a wealth of non-coding RNAs, including non-coding RNA (lncRNA) and ribosomal RNA, which hold promise as biomarkers for tumor detection and treatment [108]. Alongside traditional methods like NGS sequencing and RT-PCR, advances such as ddPCR and single-cell sequencing may offer improved accuracy and sensitivity for analyzing tumor RNA in plasma–including within TEPs [109, 110]. However, standardization in TEP detection techniques, including steps like pre-analysis and analysis, is crucial for further progress (Fig. 3C).

### TEPs can be used for early detection of CRC

Early detection of tumors can improve treatment outcomes and reduce mortality rates. In 2015, TEPs RNA sequencing was conducted on 55 healthy individuals and 228 cancer patients. The accuracy of cancer diagnosis was 96% and the accuracy of identifying the primary tumor location in these six types of cancer (non-small cell lung cancer, colorectal cancer, glioblastoma, pancreatic cancer, hepatobiliary cancer, breast cancer) was 71%. This confirmed that TEPs is a robust pan-tumor biomarker for cancer detection with high clinical relevance. [108]. In a larger sample size, it was found that TEPs were detectable in sarcomas (*n* = 153, AUC = 0.93) [113], carcinoma of endometrium (*n* = 295, AUC = 0.975) [114], ovarian cancer (*n* = 918, AUC = 0.918) [115], and non-small cell lung cancer (*n* = 876, AUC = 0.88) [116], with high diagnostic accuracy. In 2022, 1096 blood samples from patients with stage I-IV cancer were retested based on TEPs RNA, and half of them correctly detected the presence of cancer. This indicates that TEPs-based methods can be applied in cancer screening and diagnosis [117].

Accumulating evidence indicates that TEPs are effective for early detection of colorectal cancer. Luming Xu et al. conducted a retrospective cohort study. Briefly, transcriptome sequencing was performed on platelets isolated from 132 patients with early and advanced CRC and 190 controls. Through leave-one-out cross-validation, they found that the RNA profile of TEPs may be a diagnostic marker for identifying early CRC in non-cancerous diseases, but also showed high sensitivity and specificity in distinguishing CRC stages [118].

Research suggests that changes in platelet proteins might offer potential for early detection of CRC. Studies have shown that levels of specific proteins like vascular endothelial growth factor (VEGF), basic fibroblast growth factor (FGF), platelet-derived growth factor (PDGF), and platelet factor 4 (PF4) were higher in the platelets of 35 cancer patients compared to 84 healthy individuals, as measured by a technique called enzyme-linked immunosorbent assay (ELISA) [119].

Although TEPs, CTCs, and ctDNA show promising results in early detection of cancer, the accuracy of the ctDNA special classifier for plasma ctDNA classification when distinguishing healthy subjects from cancer patients was 68.7%, and the accuracy AUC of the platelet special classifier in the test set was 97.5% [114]. This suggests that combining the detection of TEPs, CTCs, and ctDNA can improve the diagnostic accuracy of tumors.

### TEPs can be used for real-time monitoring of CRC

Tumor cells and other molecules within the bloodstream can interact with platelets, inducing changes in their characteristics. These TEPs differ from normal platelets and exhibit distinct protein and RNA profiles [118, 119]. Recent research indicates that these changes in TEPs can vary across cancer types and may play a role in tumor progression [103, 120]. Bingqi Ye et al. found that four platelet-related lncRNAs: LNCAROD, SNHG20, LINC00534, and TSPOAP-AS1 were up-regulated in platelets and serum of CRC patients, suggesting that TEPs are a potential biomarker for monitoring the development of CRC [121].

Recent studies have shown that TEPs can monitor the spread of cancer to other parts of the body. TEPs can activate the coagulation cascade via tissue factor-mediated platelet activation, leading to the formation of platelet-rich clots around CTCs. This process can protect CTCs from being cleared by the immune system and promote their survival [122]. A study by Zahra Eslami-S et al. showed that platelets exposed to CRC exhibited increased expression of genes involved in cancer invasion, such as MYC, IL33, PTGS2, PTGER2, and VEGFB [123]. This suggests that inhibiting platelets using specific drugs might hold promise for reducing tumor burden [124]. However, while TEPs have been explored for monitoring CRC progression, their accuracy may be influenced by the testing conditions and potential delays.

### TEPs are promising prognostic markers for CRC

CRC is often linked to thromboembolic events due to thrombocytosis and hypercoagulability. A high platelet count is significantly associated with suppressed invasion, metastasis, and survival among CRC patients [123, 124]. Xueqin Li et al. have found elevated TEPs can promote the growth and metastasis of CRC by binding CD62P to PSGL-1 expressed on tumor macrophages (TAMs), enhance the C5 transcription in TAMs and activate the C5a/C5aR1 axis via the JNK/STAT1 pathway. The study explored the mechanism by which the interaction between platelets and TAMs can aggravate the development of CRC and proposed a potential therapeutic strategy for CRC patients [125]. While previous research suggests that platelets can influence the course and outcome of CRC through interactions with TAMs, the precise mechanisms remain unclear. A study investigating OS and cancer-specific survival (CSS) in 78 stage II CRC patients revealed a significant correlation (p-value = 0.027) between the platelet-to-lymphocyte ratio (PLR) and the effectiveness of chemotherapy when PLR was 130. This finding suggests that platelet and PLR levels might serve as potential inflammatory markers for predicting the response to adjuvant chemotherapy in stage II CRC patients [126]. Similarly, a double-center retrospective cohort study showed that postoperative and preoperative platelet ratios can predict the prognosis of cancer patients following surgery [127].

## Conclusion

Liquid biopsy can detect CTCs, ctDNA, and TEPs, suggesting it is a useful tool for early diagnosis, screening, treatment monitoring, and prognosis prediction of CRC. CTC count has long been approved by the US Food and Drug Administration as a standard for evaluating the prognosis of CRC [49]. Therefore, counts and characterization of CTCs are potential targets for future clinical applications [128]. However, the techniques for detecting and analyzing CTCs, ctDNA, and TEPs need improvement, and the criteria for evaluating them are unclear. Standardizing detection methods is crucial for improving accuracy, repeatability, and efficiency while maintaining high sensitivity and specificity. While liquid biopsy approaches like CTCs, ctDNA, and TEPs hold promise, they are not currently considered replacements for colonoscopy. Instead, we believe in exploring the potential of combining liquid biopsy with colonoscopy to reduce invasiveness and improve patient compliance. However, current research suffers from limited sample sizes and may not fully reflect real-world clinical practice. Therefore, large-scale, well-designed prospective studies are needed to explore and validate the clinical application of liquid biopsy in colorectal cancer.

## Data Availability

No datasets were generated or analysed during the current study.
